# Book Reviews

**Published:** 1832-01

**Authors:** 


### BIBLIOGRAPHICAL NOTICES.

*Cholera; its Nature, Cause, and Treatment, and Prevention, clearly and concisely explained: with an Appendix, containing Practical Remarks on Fever and Dysentery, with which Cholera is intimately connected, and frequently combined; being the Substance of Reports made to the late Government of Poland.* By Charles Searle, Esq., of the H. E. I. Company's Madras Establishment, and lately in charge of the principal Cholera Hospital at Warsaw. *Second Edition, revised and enlarged.*—Pp. 51. Highley, London.

*Cholera; its Non-contagious Nature, and the best Means of arresting its Progress, shortly examined in a Letter addressed to the Right Worshipful the Mayor of Newcastle.* By T. M. Greenhow.—Pp. 15. Charnley, Newcastle.

*How is the Cholera propagated? The Question considered, and some Facts stated.* By an AMERICAN PHYSICIAN.—Pp. 22. Miller, London.

*An Essay on the Nature and Treatment of the Indian Pestilence, commonly called Cholera.* By HENRY PENNECK, M.D.—Pp. 40. Highley, London.

*Of Pestilential Cholera; its Nature, Prevention, and Curative Treatment.* By JAMES COPLAND, M.D., Consulting Physician to Queen Charlotte's Lying-in Hospital, &c.—8vo. pp. 174. Longman, London.

*Thoughts on the best Means of Lessening the Destructive Progress of Cholera; in a Letter addressed to the* Right Honourable Viscount MELBOURNE, *Secretary of State for the Home Department.* By JOSHUA BROOKES, F.R.S. F.L.S. &c.—Pp. 15. Richard Taylor, London.

THE first edition of Mr. SEARLE's work, in consequence of his being in Poland at the time of its publication, was published in rather an imperfect state. The author having been now enabled to superintend the work himself, much new matter has been introduced, referring more particularly to the premonitory symptoms of cholera, and its relationship to fever and dysentery. In reference to bleeding in this disease, Mr. S. states in his preface, that "he has found it necessary to qualify the advice he gave on this head in the former edition."

The symptoms of the disease are briefly yet clearly described. Mr. ORTON's statement* as to the disposition to inflammatory affections when the disease is protracted, is borne out by the experience of Mr. Searle. The immediate cause of cholera appears to Mr. S. to be of the same nature as that which ordinarily gives rise to fevers of the intermittent and typhoidal character. To develope the disease, or to render the individual susceptible of its attack, a certain predisposition must exist; and Mr. Searle "is constrained to add, that a peculiar condition or epidemic influence of the atmosphere" must exist as an influential agent. In the early treatment of the disease, before the powers of the system are much prostrated, an emetic is recommended to evacuate the stomach, whether the patient has vomited or not. "As an emetic, let the patient drink freely of warm water, or what I am of opinion is better, as it operates almost instantly, and more perfectly accomplishes the purpose, is always available, and, from its stimulant and mild aperient qualities, is useful also with these intentions, namely, a *large* tablespoonful of culinary salt dissolved in half a pint of hot water, and drank as warm as it well can be." If necessary, the dose is to be repeated. Previous to the emetic, a small quantity of blood is to be taken away, if the state of the patient appear to justify it. Mr. Searle speaks very cautiously upon this point, and objects to free depletion. He recommends the administration of calomel in twelve-grain doses, which are to be repeated every hour or two, or in smaller doses if the patient improves, until bilious stools and urine are restored.

The Appendix contains some interesting remarks respecting the connexion of cholera with fever and dysentery, and additional observations upon the treatment of the former malady, which have been suggested by the recent experience of the author.

Mr. GREENHOW is a non-contagionist; but he, like others who have before him adopted the same creed, may probably change his opinion when he has made himself better acquainted with the *facts* that bear upon the subject.

The "American Physician" confesses that cholera may be a disease communicable from body to body, and obeying, like smallpox, all the laws of a contagious disease;" "but it has occurred to him, from what he has read upon the subject, that cholera, like yellow fever, has, in truth, no common attribute of a disease strictly contagious, and that we must look for some other solu-

* Vide Review of his work in the present number.

tion of the manner in which its germs, so fatal to life, are produced and sown." He recommends the removal of patients labouring under the disease to a more healthy atmosphere, and the exclusion of the other portions of the population from infected places, until a thorough ventilation and cleansing, by means of the chloride of soda or lime, can be fully effected: and he concludes, in illustration of the propriety of this measure, by remarking that "the fact of the removal of the population, and closing up of the infected streets, having often prevented the spread of yellow fever in the transatlantic cities is worth a thousand theories on the subject."

Dr. Penneck is of opinion that the "Indian pestilence" belongs to the class of fever, and not to cholera: he is convinced, as we believe most other persons are, that the "cholera" of India and Russia is very distinct from the disease of this country to which the same name is applied. Dr. P. infers from the statements of others, for he confesses he has not seen the disease, that its essence is disordered action of the brain, producing derangement of the functions, and he advises that blood should be drawn so as to produce a great effect, and yet the smallest quantity be lost to the system. This will be best effected by the rapid abstraction of a small quantity of blood from near the seat of the disease: for this purpose he recommends the method reported in the Med. Gaz. for January 1st, 1831, by Dr. Pritchard, as practised by him in cases of hemiphlegia, hydrocephalus, and "stupor or coma occurring in the course of severe typhoid fever, and in which the patients appeared to be sinking." This method consists in making an incision completely through the scalp, for the length of four or five inches, over the sagittal suture. The edges of the wound to be separated by a row of peas, and thus to convert it into an issue. The latter part of the treatment would not be required, Dr. P. says, on the present occasion. He has practised Dr. Pritchard's plan, and observing how freely the blood was discharged, and how the headach of the patient was instantly removed, and the great ease with which the bleeding could be stopped, and how quickly the wound healed, he feels convinced that, in severe cases of cholera, this will be found more efficacious than any other method of bleeding.

Various auxilliary remedies to bleeding are mentioned: but the only one which our readers have not frequently heard of is "mercurial fumigation," which is preferred to other modes of influencing the system by mercury, on account of the rapidity of its effect, and its not increasing irritation of the mucous membrane of the stomach and bowels, which must be so likely to follow from the "heroic" doses of calomel given by many Indian practitioners. Dr. Penneck's pamphlet concludes with a few remarks on the best means of securing a thorough ventilation and cleanliness.

Dr. Copland, as far back as 1822*, published his opinion that the disease ought not to be regarded as an aggravated form of cholera; and the subsequent researches of others have verified this belief. Dr. Barry, for instance, is referred to as having stated since his arrival from St. Petersburg, and more recently from Sunderland, that the disease which he there investigated is altogether distinct from the severe forms of common cholera. After attentively considering the phenomena and nature of this malady, Dr. Copland concludes a very interesting chapter on the diagnostic characters of "pestilential cholera," by observing that neither the causes which occasion this pestilence, nor the pathological states which constitute its various grades or stages of intensity, have any connexion with those of either of the forms of cholera, whether the common *bilious* variety, or the more severe form, usually denominated *spasmodic*, the *mort de chien*, &c., and that therefore the name cholera should be discarded from all scientific descriptions of it." He views the disease, in fact, as being of modern origin and *sui generis;* and, from the various accounts of the recent epidemic which

* London Med. Repository, vol. xvii. p. 407.

have fallen into our hands, we think there are very strong, if not unanswerable, facts to support this doctrine. Instead of the designation which has hitherto been employed, Dr. Copland would give to the disease the name of Asphyxia pestilenta; and certainly this denomination very correctly points out the leading features of its severe forms, although it could not perhaps be applied with equal propriety to its milder grades.

It has been said that the malady never exhibited any proofs of infection in the East: Dr. Copland clearly shews that this statement is erroneous, and that the opinions of the Medical Boards in India confirm the belief of the contagious nature of the disease.

The description of the symptoms of the disease as they arise in its different forms and stages, which is given by Dr. C. is one of the most instructive we have seen. In the *first* and *second*, and also the most intense grades of the disease, *emetics* are recommended, "both as a means of recovering the lost balance of the circulation, and determining the fluids to the surface of the body, and as a powerful agent in exciting the paralysed nerves of organic life, and in restoring the natural secretions." Turpentine liniments, and hot turpentine fomentations to the abdomen, spine, &c. are suggested as very powerful auxiliaries to blood-letting in removing congestion and equalising the circulation. We have already given full accounts of the most important works on "Cholera," and we cannot, therefore, impose upon our readers a detailed analysis of this volume, the greater part of which consists of a summary of facts derived from the best authorities; but in addition to the valuable information collected from these sources, Dr. Copland offers many original remarks, which are well worthy of attentive consideration; and, upon the whole, his book is one of the most practically useful we have met with.

Mr. Brookes, in his letter to Lord Melbourne, points out the best means of lessening the destructive progress of the disease: he recommends that some spacious building should be appropriated for those miserable and ragged beings who inhabit various courts, alleys, &c. in the metropolis, each of which may hereafter become a focus of the most destructive nature. He also very wisely suggests that such individuals might all be clothed by disburthening the wardrobes of the rich of their superfluous articles of dress; by which sacrifice, whenever the dreadful visitation should befall us, there will be fewer materials to imbibe infectious emanations. The cheap chemical process, generally attributed to Guyton de Morveau, is recommended by Mr. Brookes for the purpose of decomposing and purifying contaminated air.

In concluding our necessarily brief notice of the various works before us, we must be allowed to express an earnest hope that no more mere compilations on the subject of cholera will be obtruded upon the public or the profession. Any additional information to that which has already been stated in various forms and modes, will, of course, be received with gratitude; but if any writer in future, who has really nothing new to communicate, should expect the public to believe that he adds another work to the already too long list, from any other motive than that of taking the chance of benefiting himself, we think he must be disappointed.

---

*Pathological Anatomy of the Brain, Spinal Cord, and their Membranes; being a condensed Description of the Morbid Appearances generally met with after Death; with Cases. Illustrated by thirteen coloured Plates.* By W. P. Cocks, Surgeon.—18mo. pp. 175. Highley, London.

This little volume contains a concise account of the symptoms and morbid anatomy which indicate the various diseases of the brain and spinal cord, together with a few briefly related cases. The plates are sufficiently well executed to give the student an idea of the pathological appearances he will meet with on dissection.

*Remarks on the Subject of Lactation: containing Observations on the Healthy and Diseased Conditions of the Breast Milk; the Disorders frequently produced in Mothers by Suckling; and numerous illustrative Cases, proving that, when protracted, it is a common Cause, in Children, of Hydrencephalus, or Water in the Brain, and other serious Complaints.* By Edward Morton, m.d. Cantab. &c.—8vo. pp. 63. Longman, London.

In our Journal for August 1827, Dr. Morton published a brief communication upon the subject of the present volume: his subsequent experience has not only convinced him that the opinions there stated were correct, but that the conclusions he then arrived at may be carried still further. We agree with Dr. Morton that, as a "general rule, at nine months after birth, the child ought to be entirely weaned;" but we differ from him when he states "that in *no instance* should he be permitted to suck more than ten." We are perfectly aware of the injuries that occasionally arise, both to the mother and her infant, from long-protracted suckling, but there are certainly many cases where the positive axiom of Dr. M. could not with propriety be acted upon. The mother will frequently be able to suckle her offspring for a longer period than ten months without any injury to herself, and the state of the child's health may be such as to render weaning improper. Upon this subject we fully coincide with Dewees that any rule for weaning founded exclusively upon the age of the child, must be of very doubtful application, if not injurious in its observance.

The remarks offered by Dr. Morton on the evil effects of suckling upon women who are naturally very delicate, or who are labouring under disease, are, upon the whole, we believe, correct; but we cannot admit that the occurrence of pregnancy always renders it necessary to wean the child. "Should," says Dr. M., "a woman with an infant at the breast again become pregnant, one of two things will usually take place, either she will miscarry, or her milk will become impoverished in quality, and diminished in quantity." This statement is supported by an ingenious and a plausible argument, but, from the facts which have fallen under our own observation, we are inclined to doubt its accuracy. It may, or may not, be proper to remove the child from the breast of a pregnant nurse. If the milk disagree with the infant, it should be weaned; but if it does not, the mere existence of pregnancy does not, in our opinion, render weaning necessary.

The principal object of this work is to point out certain facts which we believe have, at least, the merit of novelty, namely, first, "that, if children be suckled for an undue length of time, they will be liable, in consequence, to be affected with inflammation of the investing membranes of the brain; secondly, that, should they not become affected with the disorder in question, during or soon after the time they are thus improperly suckled, they will nevertheless acquire therefrom a predisposition to cephalic disease, at some future period of their lives; 3dly, that children who are suckled for an undue length of time, when labouring under other diseases, will be much more liable to have the head secondarily affected than children brought up in a different manner; 4thly, that the same effects will take place in infants, if suckled by women who have been delivered for a larger period than nine or ten months, although the infants themselves may not have been at the breast for too long a period."

In illustration of these views, fifty-two cases of meningitis are briefly referred to, in all of which the children had been suckled for a longer time than Dr. Morton deems prudent; but whether, in these instances, the disease was really produced by the protracted lactation, is a question we cannot venture to determine. The subject is certainly worthy of attention. Beyond nine or ten months is considered an undue length of time by Dr. Morton.

